# Characterization of a DNA Adenine Methyltransferase Gene of *Borrelia hermsii* and Its Dispensability for Murine Infection and Persistence

**DOI:** 10.1371/journal.pone.0155798

**Published:** 2016-05-19

**Authors:** Allison E. James, Artem S. Rogovskyy, Michael A. Crowley, Troy Bankhead

**Affiliations:** 1 Paul G. Allen School for Global Animal Health, Washington State University, Pullman, Washington, United States of America; 2 Department of Veterinary Microbiology and Pathology, Washington State University, Pullman, Washington, United States of America; University of Kentucky College of Medicine, UNITED STATES

## Abstract

DNA methyltransferases have been implicated in the regulation of virulence genes in a number of pathogens. Relapsing fever *Borrelia* species harbor a conserved, putative DNA methyltransferase gene on their chromosome, while no such ortholog can be found in the annotated genome of the Lyme disease agent, *Borrelia burgdorferi*. In the relapsing fever species *Borrelia hermsii*, the locus *bh0463A* encodes this putative DNA adenine methyltransferase (*dam*). To verify the function of the BH0463A protein product as a Dam, the gene was cloned into a Dam-deficient strain of *Escherichia coli*. Restriction fragment analysis subsequently demonstrated that complementation of this *E*. *coli* mutant with *bh0463A* restored adenine methylation, verifying *bh0463A* as a Dam. The requirement of *bh0463A* for *B*. *hermsii* viability, infectivity, and persistence was then investigated by genetically disrupting the gene. The *dam*^*-*^ mutant was capable of infecting immunocompetent mice, and the mean level of spirochetemia in immunocompetent mice was not significantly different from wild type *B*. *hermsii*. Collectively, the data indicate that *dam* is dispensable for *B*. *hermsii* viability, infectivity, and persistence.

## Introduction

Relapsing fever is an arthropod-borne disease caused by several species of spirochetal bacteria in the genus *Borrelia*. While tick-borne relapsing fever is found in many endemic foci worldwide, the burden of disease is most substantial in Africa where it ranks among the top ten causes for hospitalization in Ethiopia, and among the top ten causes of mortality in children under five in Tanzania [1, 2]. Several species of relapsing fever are found in North America and cause sporadic illness, including the well-characterized species *Borrelia hermsii* [3]. The pathogenesis of relapsing fever *Borrelia spp*. is distinct from that of the closely related agent of Lyme disease, *Borrelia burgdorferi* [4, 5]. Relapsing fever *Borrelia spp*. cause acute recurrent febrile events that correspond to episodic high levels of bacteremia, whereas *B*. *burgdorferi* rapidly migrate out of the bloodstream and into host tissues that results in various clinical syndromes including arthritis, neuropathy, and carditis [6, 7]. *B*. *burgdorferi* and relapsing fever *Borrelia spp*. share many orthologous genes and exhibit significant synteny on their chromosomes [8, 9]. Additionally, many genes are unique to either Lyme disease-type or relapsing fever-type agents. The *bh0463A* gene of *B*. *hermsii* codes for a putative DNA adenine methyltransferase (Dam), and is one example of a gene found only in relapsing fever-type spirochetes [9].

DNA methylation is a crucial component of cellular defense, protecting host cells from foreign invasion of DNA and restriction modification complexes. Methylation also serves a role in the coordination and regulation of numerous cellular events, including the timing of DNA replication, partitioning of newly synthesized DNA, and DNA repair processes [10, 11]. In bacteria, methylation of the N^6^ position of adenine is critical for epigenetic gene regulation. Dams mediate the methylation of adenine in the 5’-GATC-3’ sequence shortly after DNA replication. GATC sites on the newly synthesized strand are briefly unmethylated, allowing DNA-binding proteins with affinity for the resultant hemi-methylated DNA to prevent further modification at the GATC site [10]. Other DNA-binding proteins have an affinity for fully methylated DNA, or for non-methylated DNA [11]. The differential DNA binding affinities of various proteins results in transcriptional regulation of many genes. In multiple pathogens, epigenetic regulation by way of methylation is critical for colonization in various host microenvironments, and for survival in the presence of the host’s immune response.

The role of Dam in virulence is increasingly being elucidated in other pathogens [10, 12]. In *Salmonella enterica*, *dam*^*-*^ strains have exhibited dysregulation of *in vivo*-expressed genes, reduced ability to adhere to and invade intestinal epithelium, reduced secretion of effector proteins via a type III secretion system, and membrane instability [13–16]. As a result, *Salmonella enterica dam*^*-*^ are avirulent *in vivo* [14, 15]. Likewise, certain *dam*^*-*^ strains of *Haemophilus influenzae* demonstrated a reduced ability to adhere to endothelial cells, a reduced capability for cellular invasion, and defective intracellular replication [17]. *In vivo* attenuation was also observed during murine infection with *dam*^*-*^ strains of *Yersinia pestis* and *Yersinia pseudotuberculosis* [18, 19]. In cases where Dam is essential for viability, Dam over-producing (Dam^OP^) mutants have been generated to evaluate the effects of methylation on virulence. Dam^OP^ strains of *Vibrio cholerae*, certain strains of *Yersinia pseudotuberculosis*, and *Pasteurella multocida* have also demonstrated *in vivo* attenuation [20, 21].

A few decades ago, researchers noted the presence of an adenine-specific methylation system in relapsing fever *Borrelia spp*., but not in most strains of *B*. *burgdorferi*, after digestion of genomic DNA with adenine methylation-sensitive restriction enzymes [22, 23]. While a putative adenine-specific methyltransferase was annotated in the published *B*. *hermsii* chromosome, no studies have verified the function of this predicted gene. Moreover, a role for Dam in the pathogenesis of *Borrelia spp*. has not been evaluated to date. The well-documented role for Dam in the virulence of other pathogens, and the fact that Dam was identified in relapsing fever *Borrelia spp*. but not in *B*. *burgdorferi*, led us to hypothesize that Dam is dispensable for *B*. *hermsii* viability and has a role in spirochetal pathogenicity during mammalian infection. In this report, the putative *B*. *hermsii* Dam locus (*bh0463A*) was cloned into a *dam*^*-*^ *Escherichia coli* strain. Digestion with methyl-group sensitive restriction enzymes verified the Dam function of *bh0463A*. A *dam*^*-*^ mutant clone of *B*. *hermsii* was then successfully generated, demonstrating that *bh0463A* is dispensable for *B*. *hermsii* viability. The *dam*^*-*^ mutant clone was capable of not only infecting immunodeficient mice, but could also successfully establish persistent infection in immunocompetent mice.

## Materials and Methods

### Ethics statement

The experimental procedures involving strains of inbred mice were carried out in accordance with the American Association for Accreditation of Laboratory Animal Care (AAALAC) protocol and the institutional guidelines set by the Office of Campus Veterinarian at Washington State University (Animal Welfare Assurance A3485-01 and USDA registration number 91-R-002). Washington State University AAALAC and institutional guidelines are in compliance with the U.S. Public Health Service Policy on Humane Care and Use of Laboratory Animals. Inbred mice were maintained at Washington State University (Pullman, WA, USA) in an AAALAC-accredited animal facility. The Washington State University Institutional Animal Care and Use Committee reviewed and approved the animal protocols associated with the current studies.

Mice were monitored every 24 hours by lab animal care technicians, as well as every 24 hours post infection when investigators were performing phlebotomy. Animal health was assessed by observing the general appearance of mice as well as their activity prior, during, and after handling. Mice that displayed reduced activity after moving the cage, reduced withdrawal of the hindlimb during bleeding, or had a rough haircoat were considered clinically affected. No animals became severely ill (critically depressed or moribund) or died before or during the experimental endpoint. All animals were euthanized with carbon dioxide, followed by the secondary method of cervical dislocation.

### Bacterial strain and culture conditions

The isolation and characterization of *B*. *hermsii* DAH has been described [24, 25], and was acquired as a gift from George Chaconas, who obtained it from Tom Schwan. All wild type and mutant clones of *B*. *hermsii* were cultivated at 35°C under 1.5% CO_2_ in modified Barbour-Stoenner-Kelly medium (BSK-II) supplemented with 12% rabbit serum (Accurate Chemical and Scientific Corp., Westbury, NY) [26]. Selection of transformants and growth of the *bh0463A* mutant clone was achieved with the addition of gentamicin (40 μg/mL). For *in vitro* growth assays, *B*. *hermsii* was grown to late log phase and subcultured in triplicate to a cell density of 5 X 10^5^ spirochetes per mL. Spirochetes were enumerated at 24 hour-intervals and expressed as mean densities with standard deviation. Cell densities and growth phase were monitored by visualization under dark-field microscopy using a Petroff-Hausser counting chamber.

### Verification of DNA adenine methyltransferase function

The plasmid pAE35, and a control plasmid pAE30, were generated to test the Dam activity of *bh0463A* following transformation into a commercially available *dam*^-^/*dcm*^-^ *Escherichia coli* strain (New England Biolabs, Ipswich, MA). To generate pAE30, the 135 bp *Borrelia hermsii* DAH *flgB* promoter (*flgBp*) was amplified as previously reported [27] with primers P233 and P255 that possessed NdeI and HindIII sites, respectively (Table 1). The amplification product was then cloned upstream of an *aphI gene* conferring kanamycin resistance (*kan*^R^) on the pBSV2 [28] shuttle vector using the same restriction enzymes (New England Biolabs, Ipswich, MA). The resultant *flgBp*/*kan*^R^ locus on pBSV2 was then amplified with P246 and P287, and cloned into the commercially available pJet1.2/blunt vector (Thermo-Fisher Scientific, Waltham, MA). The final control plasmid pAE30 was verified by DNA sequencing.

**Table 1:** Oligonucleotides used in this study.

Primer	Sequence (5’ to 3’)^a^
P218	CATTTCCTA**GCTAGC**TTAGGTGGCGGTACTTGGGTC
P233	ATT**CATATG**AACACCCTCTATATCACAAATT
P246	GCTAGCGCTCTGCCAGTGTTACAACC
P255	GTT**AAGCTT**GTTAAAGAAAATTGAAATAAACTTG
P287	GACGCCGGCATTACGAATTCGAGCTCGGT
P308	TAGGCCGGCACTAGTGCAAGAGCTATAGCATTAAGAGAATGGCTAAAG
P309	TAGGCCGGCTCCCCAGTGCTTCTTTTCCTTCCAACATC
P408	CCGG**GGATCC**TCTAGAGTCG
P426	TAGGGCGCGCCGTGGTTCTACAGAACCAAAAGTAGCTGCT
P427	TAGCTCGAGGGTCTCTTTAGCAGTCTCTGCCC
P428	TAG**GGATCC**AAAGGAGTATTAAATCTCCCATCTC
P429	TAG**GCTAGC**TGGTAAATATAAAAAGATTAGTCTTTATGA
P780	CATGCGAGCCTTCTAAACTACTAAATTTAC
P825	GAAGCAGATGTTTATAAAGTCAATAACTTATATC
P846	TAG**GGTACC**TTAGAAAAACTCATCGAGCATC
P847	TAG**GGTACC**GAGGGAGGTTTCCAT ATGAGCGTTGCAATACG
P848	TAGGACGTCCATCTTTACGCCTATCCAC

^a^ Restriction sites are underlined and shown in bold.

To generate the *dam* complementation plasmid, pAE35, the putative *dam* locus *bh0463A* was amplified with P847 and P848 producing an 892 bp product. No native promoter or ribosomal binding site (RBS) could be identified in the immediate upstream region of *bh0463A*. As such, P847 included a 5’ RBS and a KpnI restriction enzyme linker. The *flgBp*/*kan*^R^ amplicon was generated using P255 and P846, the latter possessing a KpnI site. Both PCR products were cleaned using a PCR cleanup kit (Qiagen, Valencia, CA) and digested with KpnI (New England Biolabs). Following ligation of the two amplicons, a single *kan*^R^-*bh0463A* construct driven by the *B*. *hermsii flgB* promoter was ligated to pJet1.2/blunt in a separate reaction. The final pAE35 construct was verified by DNA sequencing.

Both plasmids were transformed into a commercially available *dam*^-^/*dcm*^-^ *E*. *coli* strain (New England Biolabs). Following plasmid extraction (Mini Kit, Qiagen), 300 ng of either plasmid (pAE30 or pAE35) were digested with DpnI, MboI, or Sau3AI (New England Biolabs). The resulting DNA fragments were resolved with electrophoresis on a 1.8% agarose gel.

### Vector construction

The plasmid vector, pMTKO, was constructed for the stable integration of an *aacC1* gene conferring resistance to gentamicin (*gent*^*R*^) into the DNA adenine methyltransferase gene at chromosomal locus *bh0463A* (GenBank Accession Number NC_010673.1). A 2,085 base pair PCR amplicon of the *bh0463A* locus was produced with primers P426 and P427 (Table 1), and cloned into the pJET1.2/blunt vector backbone (Thermo-Fisher Scientific) to generate the plasmid pJET1.2/MT. Inverse PCR of pJET1.2/MT was performed using P428 and P429, which possess BamHI and NheI 5’ restriction sites, respectively. Amplification of the *B*. *hermsii* DAH *flgB* promoter (*flgBp*) was performed as described above, and cloned upstream of *gent* on pBSV2G [29]. The 695 base pair *flgBp-gent* construct was then amplified from pBSV2G using P408 and P218, which was cloned into the inverse PCR amplicon at the BamHI and NheI restriction sites. Sequencing to verify the appropriate insert was performed using the standard pJET1.2/blunt primers. The final construct, pMTKO, was propagated in *E*. *coli* DH5α cells, and purified using a plasmid midikit (Qiagen).

### Transformation and mutant characterization

Preparation of electrocompetent *B*. *hermsii* DAH cells and transformation were carried out as described previously for *B*. *burgdorferi* [30, 31]. After electroporation, cells were immediately placed in 5 mL pre-warmed BSK-II supplemented with 12% rabbit serum, and incubated for 24 hours at 35°C with 1.5% CO_2_. Culture volume was then increased to 50 mL with fresh medium and the transformant pool was allowed to expand in a polyclonal pool in BSK-II medium under selection with gentamicin (40 μg/mL) until the cell density reached approximately 1x10^8^ spirochetes per mL. Clonal populations were obtained via 10-fold serial dilution until the cell density was approximately 1 spirochete per mL. Two hundred μL of the resulting diluted sample was plated in each well of a 96-well plate. Plates were incubated as described above. Wells containing potential transformant clones were identified by media color change. Transformant clones were PCR screened for the presence of *gent*^*R*^, and PCR-positive clones were inoculated into 100 mL of fresh BSK-II under gentamicin selection in preparation for DNA extraction (Midi Kit, Qiagen). DNA from clones of *Bh*Δ*dam* were subjected to PCR using P825 and P780 primers, producing a 2,641 base pair amplicon that spans both upstream and downstream of the disrupted *bh0463A* locus. The resultant product was purified with a PCR cleanup kit (Qiagen), and the insertion site was verified via sequencing. The absence of Dam activity in the *bh0463A* mutant was verified by digesting 500 nanograms of genomic DNA extracted from wild type and mutant *B*. *hermsii* with either DpnI, MboI, or Sau3AI. Resultant fragments were resolved with electrophoresis on a 0.7% SeaKem LE Agarose gel (Lonza, Basel, Switzerland).

### Murine infection

Male, C.B-17/IcrHsd-*Prkdc*^*scid*^ (SCID) or C57/BL6NHsd (B6) mice of 4–6 weeks of age were purchased from Harlan (Indianapolis, IN). Both murine strains have been previously used for infection experiments involving *B*. *hermsii* [32–34]. The animals were inoculated with a *B*. *hermsii* clone via subcutaneous injection within the interscapular region at 1x10^6^ total spirochetes per mouse, except for the infectious dose experiments when each mouse was inoculated with approximately 1x10^5^, 1x10^4^, 1x10^3^, 1x10^2^, or 10 spirochetes. *B*. *hermsii* clones were passaged no more than two times *in vitro* from frozen glycerol stocks prior to murine inoculation, and both the mutant and wild-type inocula were composed of clonal populations of serotype VlpA7. In order to confirm infection, blood was drawn from a mouse via maxillary or saphenous bleed each day over the course of 3 or 10 days. Blood (10 μl) was cultured in 1 ml of BSK-II containing *Borrelia* antibiotic cocktail (0.02 mg ml^-1^ phosphomycin, 0.05 mg ml^-1^ rifampicin and 2.5 mg ml^-1^ amphotericin B). Blood cultures were incubated at 35°C under 1.5% CO_2_ for 3–4 weeks. The blood cultures were periodically examined via dark-field microscopy for the presence of *Borrelia* cells. A mouse whose blood sample(s) showed viable spirochetes via blood culture was considered infected.

### Verification of the stable disruption of *dam* and antigenic variation

The stable integration of *gent*^R^ into *bh0463A* was verified by PCR amplification and restriction enzyme digest of mutant spirochetes recovered from mice. One mL blood cultures from mice at day 10 post inoculation were verified positive by microscopy, and DNA was extracted with the Wizard Genomic DNA Kit (Promega, Madison, WI) using the manufacturer’s instructions for Gram negative bacteria. Recovered genomic DNA was then subjected to restriction enzyme digestion with DpnI and MboI, and resultant fragments were electrophoretically resolved on a 1.8% agarose gel.

The same DNA extracted from day 10 post inoculation blood cultures was used as template in a PCR reaction with primers P308 and P309 to amplify the variable major protein expression site. The resultant amplicon was resolved on a 1% agarose gel, and DNA was extracted using the QIAquick Gel Extraction Kit (Qiagen). Extracted DNA was sequenced; results were compared with the infecting serotype and analyzed on BLAST (http://blast.ncbi.nlm.nih.gov) to verify antigenic switching.

### Quantification of spirochetemia

Quantitative PCR (qPCR) of *B*. *hermsii* from mouse blood was demonstrated in a previous study to correlate with microscopic counting of spirochetes on a Petroff-Hausser counting chamber [35]. The qPCR protocol described herein was adapted from [35] and [36]. Total DNA was extracted from 40 μL of mouse blood on days 3, 7, and 10 post inoculation using the QIAamp DNA Micro Kit (Qiagen) following the handbook protocol for small volumes of blood. DNA was eluted in 20 μL of distilled water, followed by dilution 1:10 with water. Five μL of the diluted samples, 10 μL of 2X SsoAdvanced Universal SYBR Green Supermix (Bio-Rad Laboratories, Hercules, CA), and 0.4 μL each of primers P677 and P678 (5’-TAACACACCAGCATCACTAGCT, 5’-CCTTCCTCTTGCTGTCCTATCT, respectively; concentration 18.75 μM) were added to the 20 μL qPCR reaction volume. The 177 base pair product of P677 and P678 originates in the *B*. *hermsii* DAH *flaB* gene. Cycling parameters were one cycle at 98° for 2 minutes, followed by forty cycles at 98° for 5 seconds and 62° for 25 seconds. Reactions were run on the CFX96 Touch Real-Time PCR System (Bio-Rad). A standard curve was generated using the *flaB* PCR amplicon cloned into the vector pCR4-TOPO (Invitrogen, Carlsbad, CA), diluted from 10^6^ to 10 copies per 5 μL. The mean copy number was obtained from triplicate measurements of both standards and samples, and multiplied by a factor of 1000 to quantify spirochetes per mL of blood. The multiplication factor was obtained because DNA in 5 μL undiluted sample = DNA in 10 μL blood; 10 μL blood x 100 = spirochetes in 1 mL x 10 (sample dilution factor) = 10 X 100 = 1000.

### Statistical analysis

The SigmaPlot program (version 11, Systat Software, San Jose, CA) was used for all statistical analyses, except for linear regression analysis of growth curves where Microsoft Excel (version 2013) was used. Student’s t-test was used to compare the slopes of the linear regression lines (growth curves) and when comparing the means of two groups (qPCR data). Fisher’s Exact Test was used in cases where data could be applied to contingency tables (murine infection data). P-values of <0.05 were considered statistically significant.

## Results

### Verification that *bh0463A* encodes a putative DNA adenine methyltransferase

The presence of a DNA adenine-specific methylation system in relapsing fever *Borrelia spp*. was discovered several decades ago by digesting genomic DNA with adenine methylation-sensitive restriction enzymes DpnI and MboI [22, 23]. A later publication by Lescot et al. identified a putative adenine-specific methyltransferase in the chromosome of *Borrelia duttonii* and *B*. *recurrentis* [9]. For the present study, a BLAST analysis of the putative *B*. *duttonii* adenine-specific methyltransferase (*bdu_467*; GenBank Accession Number NC_011229.1) identified a highly similar gene annotated as a ‘DNA methyltransferase’ in the chromosome of *B*. *hermsii* at locus *bh0463A* (Accession Number NC_010673.1). The same BLAST analysis also identified highly similar genes in several other species of relapsing fever (*B*. *turicatae*, *B*. *parkeri*, *B*. *coriaceae*, *B*. *hispanica*, *B*. *miyamotoi*, *B*. *crocidurae*, and *B*. *persica*), indicating that the putative adenine-specific methyltransferase is conserved among relapsing fever *Borrelia* species. Further characterization of this locus on the Restriction Enzyme Database website (http://rebase.neb.com, New England Biolabs) concurred with the BLAST analysis, and identified the predicted recognition sequence of the putative methyltransferase as GATC.

**Fig 1:**
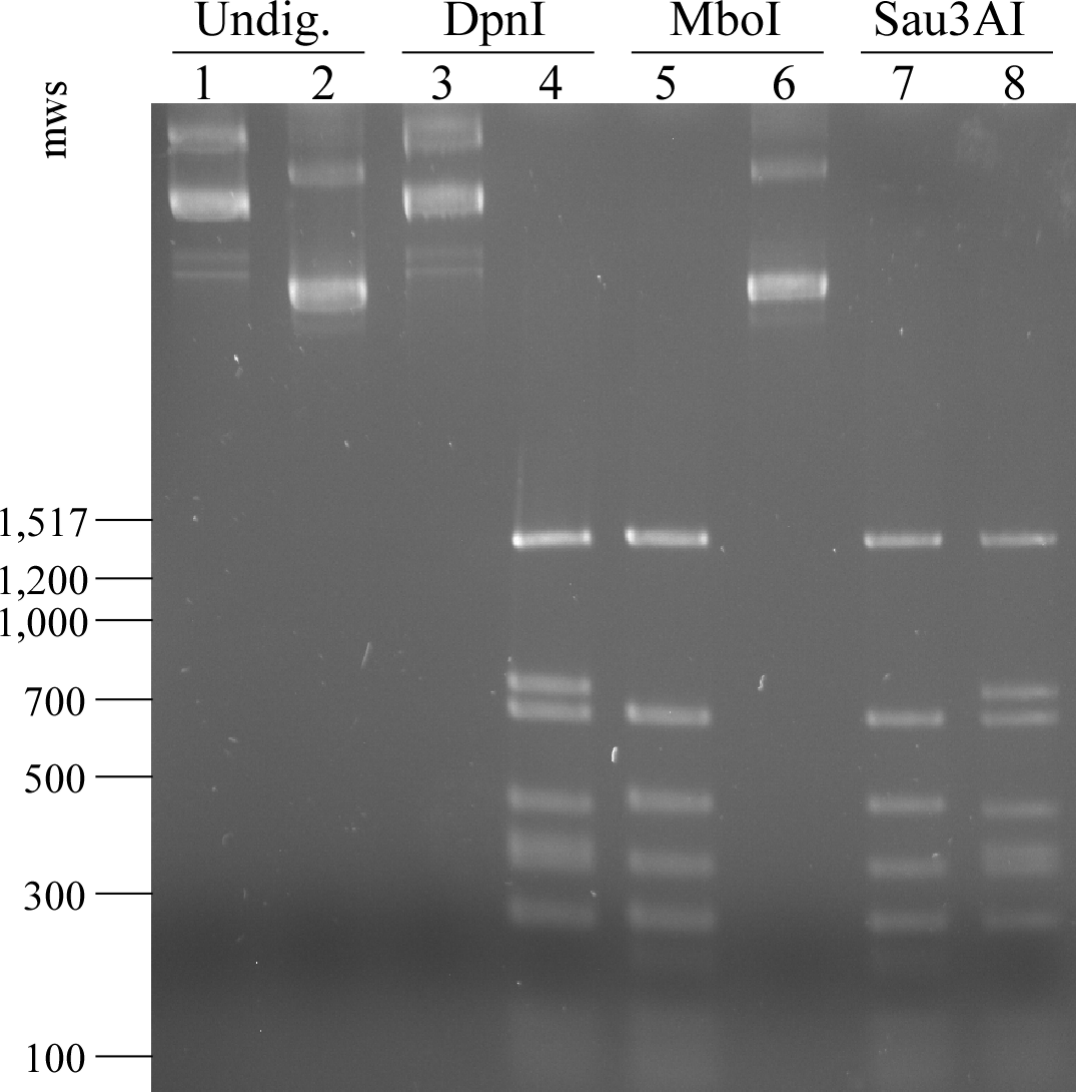
Complementation of *bh0463A* in a *dam*^-^ *Escherichia coli* strain results in adenine methylation of plasmid DNA. Control plasmid pAE30 (lanes 1, 3, 5, and 7) and a *bh0463A* complementation plasmid (pAE35; lanes 2, 4, 6, and 8) were extracted from a *dam*^-^ *E*. *coli* strain and subjected to digestion with DpnI (lanes 3 and 4), MboI (lanes 5 and 6), or Sau3AI (lanes 7 and 8). DpnI, MboI, and Sau3A1 cut GATC sequences that are adenine methylated, adenine non-methylated, or cytosine non-methylated, respectively. Sizes of selected molecular weight standards (mws) of 100 bp DNA ladder (New England Biolabs) are shown on the left in base pairs.

To verify the function of *bh0463A* as a DNA adenine methyltransferase, the predicted *dam* gene was PCR amplified and cloned downstream of a *B*. *hermsii flgB* promoter-driven kanamycin resistance cassette (*kan*^R^) in a pJet plasmid. This construct, pAE35, was then transformed into a *dam*^-^ *E*. *coli* strain. Likewise, a similar plasmid possessing only *kan*^R^, pAE30, was transformed as a control. Plasmid DNA extracted from the *dam*^-^ *E*. *coli* transformed clones was subjected to digestion with either DpnI, MboI, or Sau3AI; DpnI cuts GATC sites in which the adenine residue is methylated, MboI cuts unmethylated GATC sites, and Sau3AI cleaves all GATC sites regardless of adenine methylation, but will not cut when cytosine is methylated. The results demonstrated that, unlike the control plasmid, expression of *bh0463A* in the *dam*^-^ *E*. *coli* cells produced a restriction fragment profile indicative of adenine methylation at GATC sites (i.e. permissive digestion by DpnI and Sau3AI only; Fig 1). These results provide strong evidence for the function of *bh0463A* as a DNA adenine methyltransferase. Hereafter, the *bh0463A* locus will be referred to as *dam*.

### Generation and *in vitro* characterization of a *dam* mutant of *B*. *hermsii*

**Fig 2:**
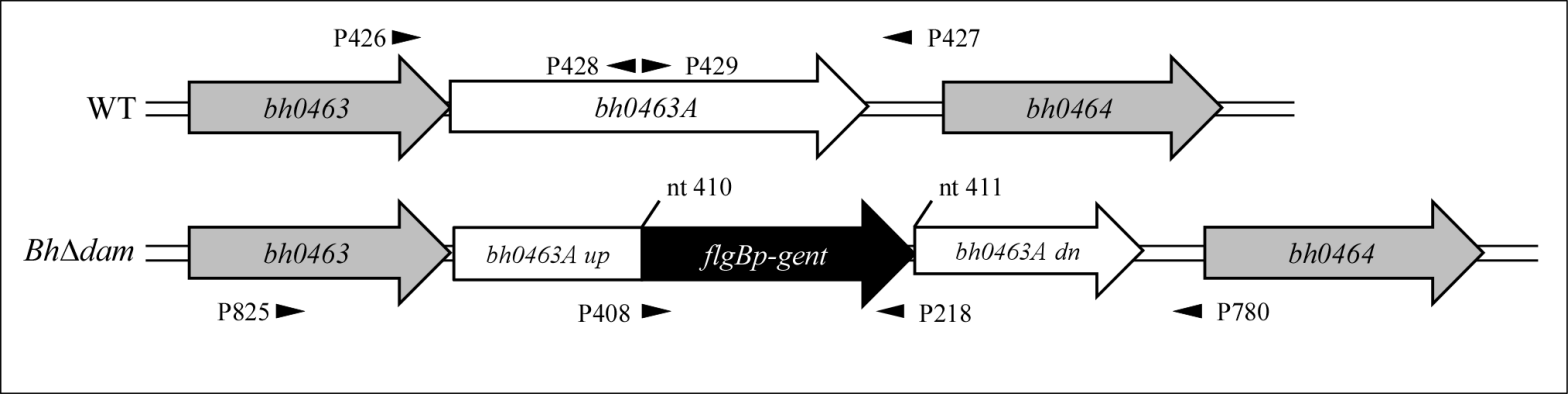
Schematic of the DNA adenine methyltransferase locus (*bh0463A*) in *B*. *hermsii* wild type (WT) and *Bh*Δ*dam*. The locations of primers used to generate pMTKO and verify *Bh*Δ*dam* are depicted as arrowheads (see Materials and Methods). nt, nucleotide; up, upstream sequence; dn, downstream sequence.

In order to determine whether Dam is dispensable for viability in *B*. *hermsii*, a mutant clone with a disrupted *dam* gene (*Bh*Δ*dam*) was generated. To achieve this, a plasmid (pMTKO) was constructed that contained a *B*. *hermsii flgB* promoter-driven *aacC1* gene conferring resistance to gentamicin (*gent*^*R*^) inserted within a copy of *bh0463A* at nucleotide position 410 (Fig 2). Electrocompetent *B*. *hermsii* cells were transformed with the pMTKO construct, and resultant transformants were isolated under gentamicin selection and PCR screened for the presence of *gent*^*R*^. Final verification of the mutant clone was achieved through PCR amplification and sequencing of the *gent*^*R*^ insertion site.

**Fig 3:**
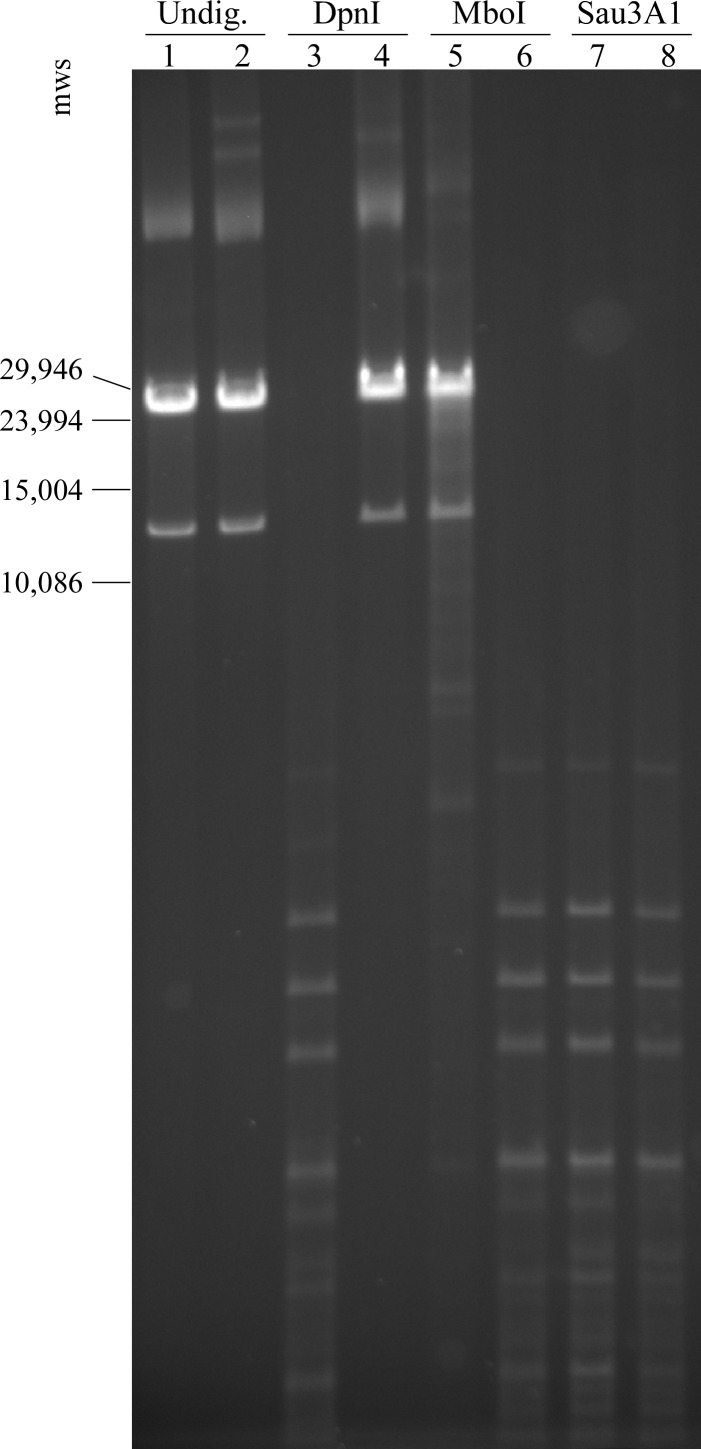
Disruption of putative methyltransferase *bh0463A* results in unmethylated genomic DNA. Lanes 1, 3, 5, and 7 contain wild type *B*. *hermsii* DNA; Lanes 2, 4, 6, and 8 contain DNA from *Bh*Δ*dam*. DNA is either undigested (lanes 1 and 2), digested with DpnI (lanes 3 and 4), MboI (lanes 5 and 6), or Sau3AI (lanes 7 and 8). DpnI, MboI, and Sau3A1 cut GATC sequences that are adenine methylated, adenine non-methylated, or cytosine non-methylated GATC sites, respectively. Sizes of selected molecular weight standards (mws) of λ DNA Mono cut mix (New England Biolabs) are shown on the left in base pairs.

To verify the disruption of Dam function in *Bh*Δ*dam*, genomic DNA from both mutant and wild type strains was subjected to restriction digest by DpnI, MboI, or Sau3AI as described above. The results demonstrated that *Bh*Δ*dam* DNA was resistant to digestion with DpnI, an enzyme requiring methylation of adenine residues for GATC cleavage, whereas wild type DNA was highly fragmented following digestion with DpnI (Fig 3). Conversely, *Bh*Δ*dam* genomic DNA is fragmented after digestion with MboI, which is blocked by adenine methylation, while wild type DNA is resistant to digestion with this enzyme. DNA isolated from both wild type *B*. *hermsii* and *Bh*Δ*dam* was equally susceptible to fragmentation by Sau3AI, indicating that the absence of *dam* in *B*. *hermsii* does not affect cytosine methylation at GATC sites. Together, the data indicate that adenine methylation is disrupted in *Bh*Δ*dam*, and provides further confirmation that *bh0463A* codes for an adenine-specific DNA methyltransferase.

**Fig 4:**
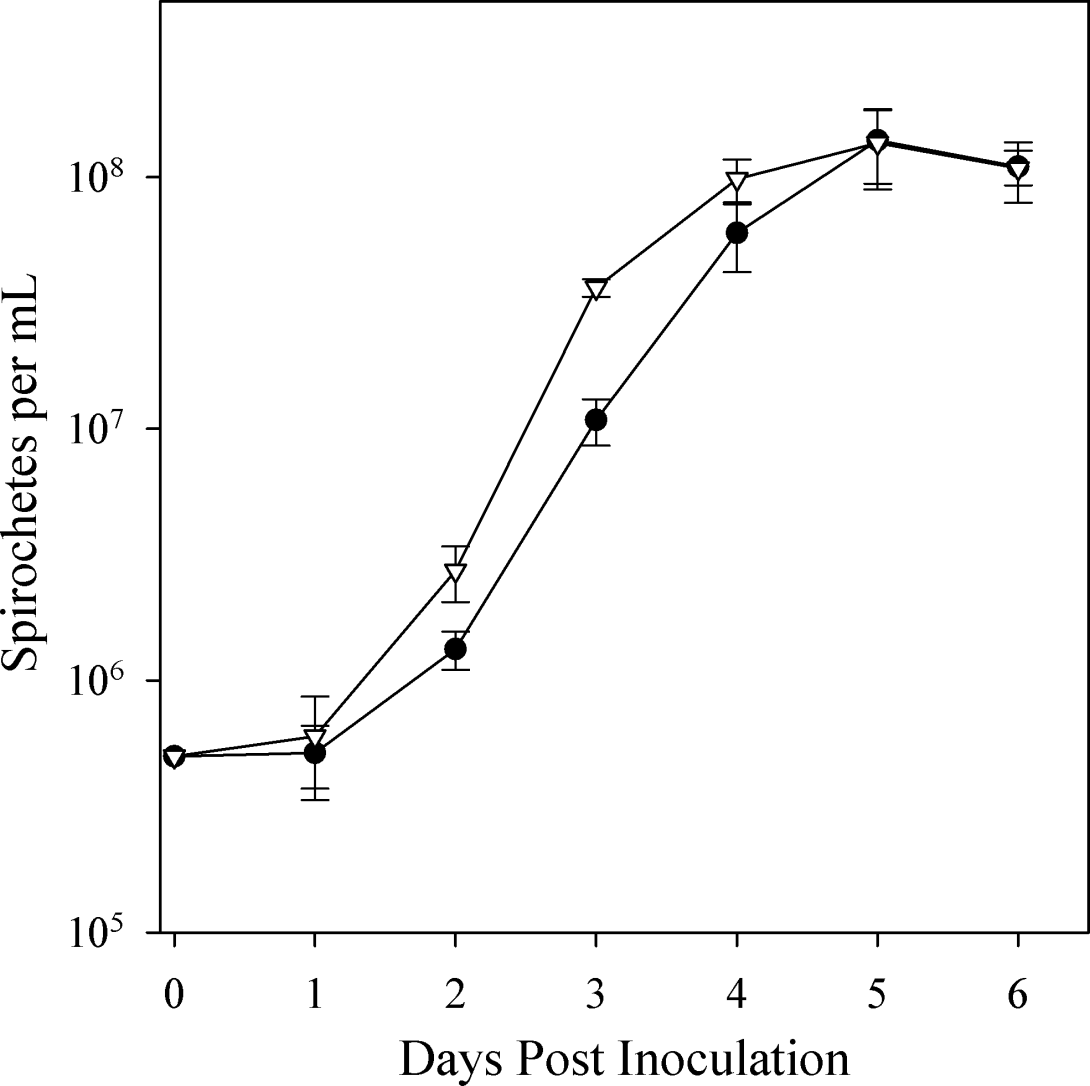
*In vitro* growth curves of wild type *B*. *hermsii* and *Bh*Δ*dam*. Wild type *B*. *hermsii* is shown with black circles; *Bh*Δ*dam* is shown with white triangles. No significant differences in growth rate or maximum cell density were detected between *Bh*Δ*dam* and wild type *B*. *hermsii*. The mean and standard deviation of triplicate measurements at each time point are plotted.

To determine whether the disruption of *dam* results in altered growth during *in vitro* cultivation, growth curves were generated for *in vitro*-grown *Bh*Δ*dam* and wild type *B*. *hermsii* (Fig 4). During the 72 hours of exponential phase growth (day 1–4 post inoculation), the *Bh*Δ*dam* doubling time was 9.8 hours while the doubling time for wild type *B*. *hermsii* was 10.5 hours. Linear regression analysis of the slopes during these three days revealed no significant differences between the growth of the mutant and wild type strains (p = 0.6). Maximum cell densities of both clones occurred at 5 days post inoculation (wild type = mean 1.4 x 10^8^, 95% confidence interval = 8.8 x 10^7^–1.9 x 10^8^; *Bh*Δ*dam =* 1.4 x 10^8^, 95% CI = 8.3 x 10^7^–1.9 x 10^8^) and were not significantly different (p = 0.9). Comparison of overall *in vitro* growth patterns between *Bh*Δ*dam* and the wild type control revealed minimal differences, demonstrating that the disruption of *dam* does not result in *in vitro* growth defects.

### *Bh*Δ*dam* is capable of persistent infection in mice

Previous studies involving *dam* mutants in other bacterial species have demonstrated a role for Dam in virulence and pathogenesis [10, 12, 37]. Thus, the ability of the *Bh*Δ*dam* mutant clone to establish persistent infection in a mammalian host was investigated. In doing so, two groups of 8 immunocompetent B6 (C57/BL6NHsd) mice each were needle inoculated with either *Bh*Δ*dam* or wild type *B*. *hermsii* at 1 x 10^6^ total spirochetes per animal. Blood was collected from each mouse every day for ten days post infection and evaluated for the presence of spirochetemia by blood culture. Thirteen immunodeficient SCID mice were also inoculated alongside immunocompetent mice with either *Bh*Δ*dam* (8 animals) or wild type *B*. *hermsii* (5 animals) to serve as an infection control. SCID mice lack antibody production, including the borreliacidal immunoglobulin M (IgM) antibodies that are rapidly produced after host infection and known to be critical for control of *B*. *hermsii* infection [38–41].

The wild type clone was able to establish infection in all 8 immunocompetent animals and were continuously detectable in most mice (7/8) throughout the ten-day period (Table 2). Similarly, *Bh*Δ*dam* was also detected in all mice by day 1 post inoculation, and could be recovered each day throughout the study period. Statistically-significant differences in the number of blood-culture positive mice between the two groups were observed only on day 8, where fewer mice produced detectable wild type spirochetemia. The finding that spirochetes were recovered in most mice on days 9 and 10, however, indicate that wild type spirochetes remained persistent throughout the study period, and that the few positive mice found on day 8 were likely a detection artifact due to low levels of spirochetemia at that particular timepoint. Similarly, for SCID mice, the results showed that 8/8 mice inoculated with the *Bh*Δ*dam* mutant clone and 5/5 mice inoculated with wild type were culture-positive for spirochetes throughout the 10 day period (Table 2), except one wild type-infected mouse that did not produce a positive culture until day 2 post inoculation.

**Table 2:** Infection by *B*. *hermsii* wild type and *Bh*Δ*dam* in B6 and SCID mice as determined by blood culture.

Day post infection	B6 mice infected with:		SCID mice infected with:	
	WT	*Bh*Δ*dam*	WT	*Bh*Δ*dam*
1	8/8	8/8	4/5^a^	8/8
2	8/8	8/8	5/5	8/8
3	8/8	8/8	5/5	8/8
4	8/8	8/8	5/5	8/8
5	8/8	8/8	5/5	8/8
6	8/8	8/8	5/5	8/8
7	7/8	8/8	5/5	8/8
8	3/8	8/8 ^b^	5/5	8/8
9	6/8	8/8	5/5	8/8
10	7/8	8/8	5/5	8/8

^a^ Values listed correspond to number of positive cultures/number tested.

^b^ Statistically-significant difference between mutant and control groups as determined by Fisher’s Exact Test (p<0.05).

To ensure the stable disruption of *dam*, DNA was extracted from spirochetes recovered on day 10 post infection from immunocompetent mice and subjected to restriction enzyme analysis with DpnI and MboI (S1 Fig). Spirochetal DNA at this time point was found to lack methylation, indicating that *dam* function was destroyed throughout the study period. Finally, PCR amplification and sequencing of the variable major protein expression site of spirochetes recovered from 3 mice in each group at day 10 post infection revealed that the locus had undergone antigenic variation (S1 File). Previous studies have shown that antigenic switching in wild type *B*. *hermsii* occurs via a gene conversion event at this single, transcriptionally active locus [42–44]. Antigenic variation has also been demonstrated to be required for persistence of this pathogen in immunocompetent mammalian hosts [36]. Specifically, all 3 mice in the *Bh*Δ*dam* group were determined to have switched from the infecting serotype of VlpA7 to VlpA25. Overall, the data demonstrate that the disruption of Dam function by integration of *gent*^R^ into *dam* is stable, and that Dam is dispensable for infection and persistence in immunocompetent murine host.

**Fig 5:**
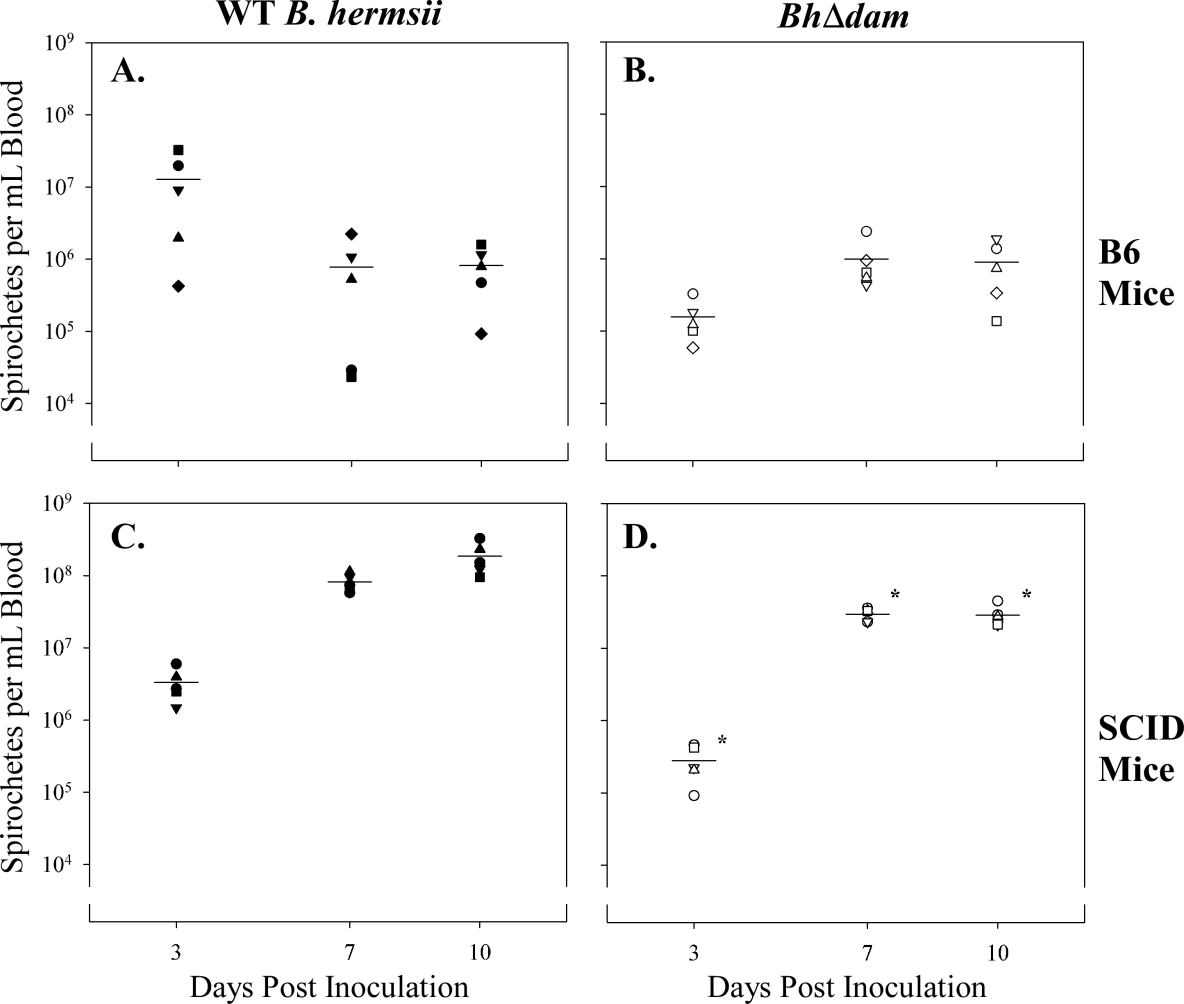
*Bh*Δ*dam* persists in the blood of B6 and SCID mice. Spirochetemia as determined by qPCR for A) wild type (WT) *B*. *hermsii* in B6 mice; B) *Bh*Δ*dam* in B6 mice; C) WT *B*. *hermsii* in SCID mice; and D) *Bh*Δ*dam* in SCID mice. Differences in spirochetemic density between strains at days 3, 7, and 10 post infection in B6 mice were not statistically significant (p>0.05). Conversely, in SCID mice, spirochetemia of *Bh*Δ*dam* at all timepoints was significantly lower than WT *B*. *hermsii* (p<0.01, denoted by an asterisk). Spirochetemia from each mouse is represented by a different shape; mean density is depicted by a line.

Despite the ability of *Bh*Δ*dam* to infect and persist in the murine host, it remained unclear whether disruption of *dam* results in an attenuation of *B*. *hermsii in vivo*. To ascertain the role of *dam* in spirochetal fitness during murine infection, qPCR quantification of spirochetemia was employed. For this assay, two groups of 5 B6 mice were infected with 1 x 10^6^ wild type *B*. *hermsii* or *Bh*Δ*dam*. Blood samples were collected on days 3, 7, and 10 post inoculation, and total DNA was extracted to be used as a template for qPCR. These time points were selected because in our hands, wild type *B*. *hermsii* typically achieves peaks of spirochetemia at days 3 and 7 post inoculation, and day 10 concludes the experiment. In B6 mice, mean spirochete density of *Bh*Δ*dam* at all three timepoints was not significantly different from wild type *B*. *hermsii* (Fig 5A and 5B, data in S1 Table). To determine whether the spirochete density of the *Bh*Δ*dam* mutant achieved similar levels as the wild type strain in the less stringent host environment of an immunodeficient mouse, blood from 5 SCID mice inoculated with wild type or mutant strains was also used as a template for qPCR on days 3, 7, and 10. Both strains produced high density spirochetemia in SCID mice, but the spirochete burden in mice infected with *Bh*Δ*dam* at all 3 time points was found to be significantly lower than those infected with wild type *B*. *hermsii* (p<0.01; Fig 5C and 5D, data inS1 Table). While qPCR results demonstrate statistically significant differences in SCID mice, the biological relevance of these results is unknown and warrants further investigation. Nevertheless, the qPCR results from both immunocompetent and immunodeficient mice demonstrate that *dam* does not impart *B*. *hermsii* with a substantial *in vivo* fitness advantage, because the spirochete density of *Bh*Δ*dam* in the blood remains grossly elevated at all three time points in both strains of mice. This, combined with the detection of spirochetes in most mice throughout the 10 day experiment, indicates that *dam* is dispensable for murine infection and persistence.

### *Bh*Δ*dam* is capable of infection at low doses

To evaluate whether *Bh*Δ*dam* retained its ability to infect mice at low doses, 5 groups of B6 and SCID mice, composed of 6 and 3 animals per group respectively, were infected with decreasing doses of wild type *B*. *hermsii* or *Bh*Δ*dam* (approximately 1 x 10^5^, 10^4^, 10^3^, 10^2^, and 10 spirochetes per inoculum). Blood samples were taken from each mouse every day until day 3 post inoculation and monitored by blood culture for the presence of spirochetes. In B6 mice, all doses of wild type *B*. *hermsii* and *Bh*Δ*dam* resulted in infection by day 2 post inoculation (Table 3). Likewise, for the SCID mice, all infectious doses produced positive spirochetemia for both wild type and *Bh*Δ*dam* throughout the 3 day study (S2 Table). The data indicate that the overall ability of *Bh*Δ*dam* to infect the murine host is independent of dose and similar to wild type *B*. *hermsii*.

**Table 3:** Infection of B6 mice with wild type *B*. *hermsii* and *Bh*Δ*dam* at decreasing infectious doses of approximately 1 x 10^5^ to 10 spirochetes.

Day post inoculation:	B6 mice inoculated with:									
	WT					*Bh*Δ*dam*				
	10^5^	10^4^	10^3^	10^2^	10	10^5^	10^4^	10^3^	10^2^	10
1	5/6^a^	4/6	6/6	6/6	5/6	5/6	3/6	2/6	2/6	0/6^b^
2	6/6	6/6	6/6	6/6	6/6	6/6	6/6	6/6	6/6	6/6
3	6/6	6/6	6/6	6/6	6/6	6/6	6/6	6/6	6/6	6/6

^a^ Values listed correspond to number of positive mice/number tested.

^b^ Statistically-significant difference between mutant and control groups as determined by Fisher’s Exact Test (p<0.05). Note that a complemented mutant was not generated for comparison (see below).

### Attempted complementation of the *Bh*Δ*dam* mutant

Despite clear evidence that disruption of the *bh0463A* gene of *B*. *hermsii* results in a loss of adenine-specific DNA methylation, gene complementation was attempted in order to verify that the difference between the infection profiles of *B*. *hermsii* wild type and *Bh*Δ*dam* were due exclusively to the disruption of *dam*. Initially, a shuttle vector was generated that would allow for *in trans* complementation using methods described in Battisti et al. [27]. Attempts to transform wild type *B*. *hermsii* with the shuttle vector resulted in either no transformants, or integration of the plasmid into the genome. In another effort, a construct was generated that would replace the disrupted *dam* gene with a wild type copy that included a downstream kanamycin resistance cassette (*kan*^*R*^) *in cis*. Two transformations were attempted; both resulted in transformants with inappropriate rearrangements at the *dam* locus. A final attempt to complement *dam in trans* on a 200 kilobase linear plasmid of *B*. *hermsii* via allelic exchange utilizing methods described in Fine et al. also failed [31]. No transformants resulted from this effort. Thus far, all attempts to complement the *Bh*Δ*dam* mutant clone have been unsuccessful.

In an effort to verify that the disruption of *dam* results in an inability to persist in the absence of a complemented mutant, a second independently-generated clone of *Bh*Δ*dam* (*Bh*Δ*dam*#2) was generated, characterized, and used to infect B6 mice and SCID mice. Transformation and verification of *Bh*Δ*dam*#2 was carried out by sequencing and restriction enzyme digestion as described for the first clone (S2 Fig). *In vitro* growth curves comparing *Bh*Δ*dam*#2 to wild type *B*. *hermsii* during 3 days of exponential growth (days 1–4 post inoculation) revealed that *Bh*Δ*dam*#2 grew significantly faster than the control strain as determined by linear regression analysis and comparison of the slopes (S3 Fig; p = 0.005). Correspondingly, the doubling time for *Bh*Δ*dam*#2 was 8.8 hours, while the doubling time for wild type was 10.2 hours. Mean maximum cell density was not significantly different between the two strains (*Bh*Δ*dam*#2 = 2.8 x 10^8^ spirochetes per mL, 95% CI = 2.1 x 10^8^–3.6 x 10^8^; wild type = 2.2 x 10^8^, 95% CI = 1.6 x 10^8^–2.7 x 10^8^; p = 0.2), although *Bh*Δ*dam*#2 reached maximum cell density on day 4 post inoculation while wild type *B*. *hermsii* peaked at day 6. Finally, B6 and SCID mice were inoculated with 1 x 10^6^ spirochetes, and infection was monitored by blood culture each day for 10 days post inoculation. Like the first clone, all SCID and B6 mice were positive throughout the 10 day experiment (S3 Table), except one SCID mouse that did not become positive until day 2. Together, the results from the second clone further support that the disruption of *dam* does not result in *in vitro* growth deficiencies, nor does it result in significant *in vivo* attenuation leading a lack of persistence. Despite the absence of a complement, two clones of *Bh*Δ*dam* exhibiting similar infection phenotypes provides confidence that Dam is dispensable for mammalian infection and persistence.

## Discussion

It has been previously reported that adenine methylation systems are found in relapsing fever *Borrelia spp*., but are absent in most *B*. *burgdorferi* strains [22, 23]. Correspondingly, a putative Dam is encoded in the chromosome of *Borrelia recurrentis* and *B*. *duttonii*, but not in *B*. *burgdorferi* [9]. For the present study, analyses with BLAST and the Restriction Enzyme Database (http://rebase.neb.com) revealed that an orthologous gene with a putative Dam function exists in *B*. *hermsii* at chromosomal locus *bh0463A* (GenBank Accession Number NC_010673.1), and is conserved in other relapsing fever-type *Borrelia*. After cloning the *B*. *hermsii* gene into a *dam*^-^ *E*. *coli* strain, extracted DNA was digested with restriction enzymes sensitive to adenine methylation. The resulting restriction fragment profile provided strong evidence that *bh0463A* is indeed an adenine-specific methyltransferase. Because of the role for DNA methylation in the virulence of other pathogens, the presence of *dam* in *B*. *hermsii* led to the hypothesis that the gene product serves a role in host pathogenicity. The findings reported here are the first to show that disruption of *dam* in a relapsing fever-type *Borrelia* species does not result in significant *in vitro* or *in vivo* attenuation, a rather surprising finding based on studies involving *dam*^-^ strains of other important pathogens [10, 12, 37].

The finding that mean spirochete density is significantly lower for *Bh*Δ*dam* at days 3, 7, and 10 post inoculation compared to the wild type strain in SCID mice is somewhat puzzling, as this difference is not observed when the mutant clone is inoculated into the more taxing environment of the immunocompetent host. Whether this finding is biologically relevant must be examined more closely, as the overall spirochete burden of the mutant strain in SCID mice was grossly elevated, as expected. The apparently subtle differences between strains, and their interactions with the host’s immune system, will be evaluated in forthcoming studies using a larger sample size and increasing the sampling frequency. Nevertheless, the results reported herein demonstrate that the *B*. *hermsii* Dam is dispensable for murine infection and persistence.

The hallmark of relapsing fever is the repeated waxing and waning of spirochetemia that parallels febrile episodes [5]. The episodic outgrowth of each peak is dominated by a single serotype of the variable major protein (Vmp) [45]. The *B*. *hermsii* genome harbors a large repertoire of non-expressed *vmp* genes that are switched via gene conversion into the single transcriptionally-active expression site during infection of a mammalian host [42, 43, 46]. The overall result of this antigenic variation system is that it provides the spirochete with the capacity to repeatedly evade the highly serotype-specific host humoral response [41, 47]. Antigenic switching is required for spirochete persistence in mice beyond the first wave of spirochetemia, which typically peaks on day 3 post infection [36, 39]. By day 10 post infection, when antigenic variation was confirmed in *Bh*Δ*dam*, it would be expected that all spirochetes in the infecting inoculum, even minor serotypes if present, would have undergone switching or be cleared. By this point, each immunocompetent mouse would be expected to have undergone 1–2 relapses [36, 39]. While antigenic switching was verified to occur in *Bh*Δ*dam*, it is not clear whether the kinetics of such switching could be mediated by Dam. Increased antigenic switch rates in *Bh*Δ*dam* may explain why the mutant was detected each day throughout the B6 murine infection experiment, while wild type *B*. *hermsii* could not be recovered on low density days. Moreover, in a report by Barbour et al. that describes a loose, programmed order for within-host antigenic variation in *B*. *hermsii*, certain serotypes appeared more frequently than others when infected with spirochetes of the serotype VlpA7 [48]. Interestingly, while *Bh*Δ*dam* underwent antigenic switching, spirochetes from all three mice switched to the same serotype (VlpA25), a serotype that was not identified with any frequency in the reported findings by Barbour et al. This raises the possibility that the absence of Dam disrupts the programmed order for antigenic switching in *B*. *hermsii*. Future studies should include a closer examination of the role for Dam in immune evasion, both in the rate and order of Vmp switching, and in mechanisms for early IgM immune escape.

The data clearly indicate that Dam is not absolutely required for infection or persistence in our murine model, but the presence of *dam* orthologs in numerous relapsing fever *Borrelia spp*. raises the question of why the gene is so highly conserved in these spirochetes. One possibility is that Dam may be essential in some component of the life cycle of *B*. *hermsii* that is not recreated in the laboratory. In nature, the spirochete must be capable of survival in diverse reservoir species, and thrive under the pressure of co-infecting pathogens and bacteriophages. In this context, even small reductions in replication rate, as was observed in B6 and SCID mice immediately post infection, may adversely affect the propagation of this pathogen. Another explanation for the conservation of Dam is that it may serve a role in the tick phase of the *B*. *hermsii* lifecycle, or in the regulation of differential gene expression as the spirochete moves between the mammalian host and tick vector. For example, in the tick phase of the *B*. *hermsii* lifecycle, *vmp* transcription is turned ‘OFF’, while another distant site required for tick transmission, *vtp* (variable tick protein), is turned ‘ON’ [36, 49]. While both Vtp and Vmp are required for the *B*. *hermsii* lifecycle, the mechanisms regulating their mutually exclusive expression remains unknown [36, 39]. The absence of the Dam gene in *B*. *burgdorferi*, however, supports that if *dam* is involved in differential gene regulation in the tick, it is uniquely required for relapsing fever spirochetes. Indeed, significant differences between the two groups exist, with many relapsing fever-type *Borrelia* being transmitted by the soft-bodied, fast-feeding *Ornithodoros spp*. ticks, while *B*. *burgdorferi* requires the hard-bodied, slow-feeding *Ixodes* tick [50, 51]. Whether *dam* is required for the tick phase of relapsing fever spirochetes will be the subject of forthcoming studies.

Ideally, the results herein would be supported by a mutant clone complemented with the *bh0463A* gene. Although complementation of *B*. *hermsii* has been successful for other groups [27, 36, 52], all attempts to generate a *dam* complement failed. Despite the published descriptions of numerous complementation mutants of *B*. *burgdorferi*, difficulties in genetic complementation of *Borrelia* have been documented [53, 54]. A major limitation of not having a complement is our inability to test whether the disruption of *dam* resulted in polar effects on adjacent genes. Polar effects, however, are unlikely due to the fact that significant phenotypic differences were not observed between wild type *B*. *hermsii* and *Bh*Δ*dam*. Moreover, both *bh0463A* and *gent*^R^ genes are transcribed in the same direction, and none of the native sequence was deleted in *Bh*Δ*dam*. Thus, the native terminator sequence is expected to terminate the mutant transcript in the same fashion as the wild type transcript, further minimizing the likelihood that polar effects had any impact on a possible mutant phenotype. Despite the lack of a complement, the absence of significant differences of two independently-generated clones of *Bh*Δ*dam* in regard to *in vitro* growth, infectivity, and *in vivo* persistence, along with restriction fragment evidence for the disruption of Dam function prior to and after murine infection, instills confidence in the validity of the results: the absence of adenine-specific DNA methylation does not result in marked phenotypic differences during mammalian infection.

The present data indicate that *dam* does not influence the ability of *B*. *hermsii* to infect or persist in the mammalian host. In addition to characterizing the role for *dam* in the arthropod portion of the lifecycle, it would also be prudent to investigate the utility of *Bh*Δ*dam* as a molecular tool. For example, the role for DNA adenine methylation in restriction modification systems and in DNA partitioning have been well established [10, 11]. Therefore, future studies will be directed toward understanding whether *Bh*Δ*dam* provides improved transformation efficiency when generating genetic mutants, and determining if the absence of Dam activity allows for *B*. *hermsii* plasmid loss. Unlike *B*. *burgdorferi*, the *B*. *hermsii* genome is stable following transformation and *in vitro* passage [31, 55, 56]. Both tools have the potential to improve the investigation of putative virulence factors, and provide insight into the evolution of various relapsing fever and Lyme *Borrelia spp*.

## Supporting Information

S1 Fig *Bh*Δ*dam* remains Dam deficient on day 10 post inoculation. Pooled *Bh*Δ*dam* DNA extracted from 5 B6 mice on day 10 post inoculation was subjected to DpnI and MboI restriction enzyme digestion to verify the stable disruption of Dam function. DpnI and MboI cleave sequences that are adenine methylated and adenine non-methylated, respectively. Sizes of selected molecular weight standards (mws) of 100 bp DNA ladder (New England Biolabs) are shown on the left in base pairs.
(TIF)

S2 Fig Verification of Dam disruption in *Bh*Δ*dam*#2 by restriction enzyme digestion. DNA from *Bh*Δ*dam*#2 is either undigested, or digested with DpnI, MboI, or Sau3AI. DpnI, MboI, and Sau3A1 cut GATC sequences that are adenine methylated, adenine non-methylated, or cytosine non-methylated GATC sites, respectively. The absence of DNA cleavage with DpnI digestion and fragmentation with MboI digestion verifies the disruption of Dam function.
(TIF)

S3 Fig *In vitro* growth curves of wild type *B*. *hermsii* and *Bh*Δ*dam*#2. Wild type *B*. *hermsii* is shown with black circles; *Bh*Δ*dam*#2 is shown with white triangles. The mean and standard deviation of triplicate measurements at each time point are plotted.
(TIF)

S1 File Partial nucleotide alignment of the *Bh*Δ*dam* variable membrane protein (*vmp*) prior to infection compared to the sequences of *vmp* from *Bh*Δ*dam* recovered from 3 mice on day 10 post infection. Through sequencing of *vmp* and BLAST analysis, the *vmp* serotype in the infecting inoculum was determined to be VlpA7. The *vmp* serotype of *Bh*Δ*dam* recovered from all B6 mice on day 10 was determined to be VlpA25.
(PDF)

S1 Table Mean spirochete density as determined by qPCR for wild type *B*. *hermsii* and *Bh*Δ*dam* at days 3, 7, and 10 post inoculation. (DOCX)

S2 Table Infection of SCID mice with wild type *B*. *hermsii* and *Bh*Δ*dam* at decreasing infectious doses of approximately 1 x 10^5^ to 10 spirochetes. (DOCX)

S3 Table Infection by a second, independently generated clone of *Bh*Δ*dam* (*Bh*Δ*dam*#2) in B6 and SCID mice as determined by blood culture. (DOCX)
