# Supplementary material for: Characterization of a DNA Adenine Methyltransferase Gene of Borrelia hermsii and Its Dispensability for Murine Infection and Persistence
Source: PLoS One. 2016 May 19;11(5):e0155798. doi: 10.1371/journal.pone.0155798 (PMC4873019; doi:10.1371/journal.pone.0155798)
Supplement: S1 File — Through sequencing of vmp and BLAST analysis, the vmp serotype in the infecting inoculum was determined to be VlpA7. The vmp serotype of BhΔdam recovered from all B6 mice on day 10 was determined to be VlpA25. (PDF) [file pone.0155798.s004.pdf]

```
        BhΔdam  vmp day 0    (268) TGTTACTGACATGAAGAGAGA---AGGAAATCCTAATGCTTCTGCAACTGAGACTGCGGTAAAAACACTAA-TTGATAATACTCTTGATAAGATA-ATAG
BhΔdam vmp Mouse 1 Day 10          TTTTGCTGCCCTTACCACACATTTAGCTTACTTTTAAAGTATTAATTATTTGATTACAATAAAAACATACTCTTAATATCACACTTGCAATTATTTATAT
BhΔdam vmp Mouse 2 Day 10          --TTTCTGCCCTTACCACACATTTAGCTTACTTTTAAAGTATTAATTATTTGATTACAATAAAAACATACTCTTAATATCACACTTGCAATTATTTATAT
BhΔdam vmp Mouse 3 Day 10          TTTTGCTGCCCTTACCACACATTTAGCTTACTTTTAAAGTATTAATTATTTGATTACAATAAAAACATACTCTTAATATCACACTTGCAATTATTTATAT

        BhΔdam  vmp day 0    (363) AAGGTGCTGAGAC-TGCAAGTGAGGCTAT-TGGTGATGCTGGTGACCCAATTGGTAAT-GTTGCTGCTGGTGGTGCTGGTGCGGG--TACAGGTGCTATT
BhΔdam vmp Mouse 1 Day 10          TCAATCTTTACTCATATAAACAATTTTATATACAAATAAACATTCCCTAAATAACATTAGAAGCTAAGAGCATTAC-GCTCCTAGCTTCCTATTTCTTTT
BhΔdam vmp Mouse 2 Day 10          TCAATCTTTACTCATATAAACAATTTTATATACAAATAAACATTCCCTAAATAACATTAGAAGCTAAGAGCATTAC-GCTCCTAGCTTCCTATTTCTTTT
BhΔdam vmp Mouse 3 Day 10          TCAATCTTTACTCATATAAACAATTTTATATACAAATAAACATTCCCTAAATAACATTAGAAGCTAAGAGCATTAC-GCTCCTAGCTTCCTATTTCTTTT

        BhΔdam  vmp day 0    (458) GGGGATGGTGTTGATAATCTAATAAATGGAATTAAGGCAATTGTAGAAGTAGTACTTAAAGAAGGGAATGCTGAGGCTGGAGATGGTAAAAAGGCCGATG
BhΔdam vmp Mouse 1 Day 10          TGACTTTATTTTTCCCTGCTCTTACAGCTTACTTAATAACTTATCTTATAATAACTTCTTATTTTTCATTACAACAATATAGAAGGCATCTGAGACGCAT
BhΔdam vmp Mouse 2 Day 10          TGACTTTATTTTTCCCTGCTCTTACAGCTTACTTAATAACTTATCTTATAATAACTTCTTATTTTTCATTACAACAATATAGAAGGCATCTGAGACGCAT
BhΔdam vmp Mouse 3 Day 10          TGACTTTATTTTTCCCTGCTCTTACAGCTTACTTAATAACTTATCTTATAATAACTTCTTATTTTTCATTACAACAATATAGAAGGCATCTGAGACGCAT

        BhΔdam  vmp day 0    (558) CTCTTGGAGCAAGAGGTGCTAATGCTGGTGATGCAGGAAAGTTATTTGGTAATACTGGTAATAATGGTGCTATTGATTCTGCAGATAATGC---AAAGAA
BhΔdam vmp Mouse 1 Day 10          CTCTAAGATTGCCTTCTACTCTTACTTTT-----AAGTAATTAATCTAACTATCTATCTTATTACTGACCTGTTGCTTTTGGAGCTCTTGCTTTATCTAT
BhΔdam vmp Mouse 2 Day 10          CTCTAAGATTGCCTTCTACTCTTACTTTT-----AAGTAATTAATCTAACTATCTATCTTATTACTGACCTGTTGCTTTTGGAGCTCTTGCTTTATCTAT
BhΔdam vmp Mouse 3 Day 10          CTCTAAGATTGCCTTCTACTCTTACTTTT-----AAGTAATTAATCTAACTATCTATCTTATTACTGACCTGTTGCTTTTGGAGCTCTTGCTTTATCTAT
```
