# Supplementary material for: Characterization of a DNA Adenine Methyltransferase Gene of Borrelia hermsii and Its Dispensability for Murine Infection and Persistence
Source: PLoS One. 2016 May 19;11(5):e0155798. doi: 10.1371/journal.pone.0155798 (PMC4873019; doi:10.1371/journal.pone.0155798)
Supplement: S1 Table — (DOCX) [file pone.0155798.s005.docx]

| Day post inoculation | B6 mice inoculated with: | | SCID mice inoculated with: | |
| --- | --- | --- | --- | --- |
|  | WT | *Bh*Δ*dam* | WT | *Bh*Δ*dam* |
| 3 | ^a^1.3 x 10^7^,  9.7 x 10^5^ – 2.5 x 10^7^ | 1.6 x 10^5^,  6.7 x 10^4^ – 2.5 x 10^5^ | 3.3 x 10^6^,  1.8 x 10^6^ – 4.8 x 10^6^ | ^*,b^ 2.8 x 10^5^,  1.4 x 10^5^ – 4.2 x 10^5^ |
| 7 | 7.8 x 10^5^,  -3.1 x 10^4^ – 1.6 x 10^6^ | 9.9 x 10^5^,  2.9 x 10^5^ – 1.7 x 10^6^ | 8.2 x 10^7^,  6.2 x 10^7^ – 1.0 x 10^8^ | *^,c^ 3.0 x 10^7^,  2.4 x 10^7^ – 3.5 x 10^7^ |
| 10 | 8.2 x 10^5^,  3.1 x 10^5^ – 1.3 x 10^6^ | 9.0 x 10^5^,  2.6 x 10^5^ – 1.5 x 10^6^ | 1.9 x 10^8^,  1.0 x 10^8^ – 2.7 x 10^8^ | *^,d^ 2.9 x 10^7^,  2.0 x 10^7^ – 3.7 x 10^7^ |

^a^ Values listed correspond to mean spirochete density, 95% confidence interval

^*^ Statistically significant difference between mutant and control groups as determined by Student’s t-Test (p<0.05)

^b^ 95% confidence interval for the difference of the means (WT-*Bh*Δ*dam*) = 1.3 x 10^6^ – 4.8 x 10^6^
^c^ 95% confidence interval for the difference of the means (WT-*Bh*Δ*dam*) = 2.8 x 10^7^ – 7.6 x 10^7
d^ 95% confidence interval for the difference of the means (WT-*Bh*Δ*dam*) = 5.9 x 10^7^ – 2.5 x 10^8^
