# Supplementary material for: Characterization of a DNA Adenine Methyltransferase Gene of Borrelia hermsii and Its Dispensability for Murine Infection and Persistence
Source: PLoS One. 2016 May 19;11(5):e0155798. doi: 10.1371/journal.pone.0155798 (PMC4873019; doi:10.1371/journal.pone.0155798)
Supplement: S2 Table — (DOCX) [file pone.0155798.s006.docx]

| Day post inoculation: | SCID mice inoculated with: | | | | | | | | | |
| --- | --- | --- | --- | --- | --- | --- | --- | --- | --- | --- |
|  | WT | | | | | *Bh*Δ*dam* | | | | |
|  | 10^5^ | 10^4^ | 10^3^ | 10^2^ | 10 | 10^5^ | 10^4^ | 10^3^ | 10^2^ | 10 |
| 1 | 3/3^a^ | 3/3 | 3/3 | 3/3 | 3/3 | 3/3 | 3/3 | 3/3 | 3/3 | 3/3 |
| 2 | 3/3 | 3/3 | 3/3 | 3/3 | 3/3 | 3/3 | 3/3 | 3/3 | 3/3 | 3/3 |
| 3 | 3/3 | 3/3 | 3/3 | 3/3 | 3/3 | 3/3 | 3/3 | 3/3 | 3/3 | 3/3 |

^a^ Values listed correspond to number of positive mice/number tested.
